# Pathogen Quantitation in Complex Matrices: A Multi-Operator Comparison of DNA Extraction Methods with a Novel Assessment of PCR Inhibition

**DOI:** 10.1371/journal.pone.0017916

**Published:** 2011-03-23

**Authors:** Alessandra Pontiroli, Emma Rachel Travis, Francis Patrick Sweeney, David Porter, William Hugo Gaze, Sam Mason, Victoria Hibberd, Jennifer Holden, Orin Courtenay, Elizabeth Margaret Helen Wellington

**Affiliations:** University of Warwick, School of Life Sciences, Coventry, United Kingdom; Fundació Institut Germans Trias i Pujol; Universitat Autònoma de Barcelona CibeRES, Spain

## Abstract

**Background:**

*Mycobacterium bovis* is the aetiological agent of bovine tuberculosis (bTB), an important recrudescent zoonosis, significantly increasing in British herds in recent years. Wildlife reservoirs have been identified for this disease but the mode of transmission to cattle remains unclear. There is evidence that viable *M. bovis* cells can survive in soil and faeces for over a year.

**Methodology/Principal Findings:**

We report a multi-operator blinded trial for a rigorous comparison of five DNA extraction methods from a variety of soil and faecal samples to assess recovery of *M. bovis* via real-time PCR detection. The methods included four commercial kits: the QIAamp Stool Mini kit with a pre-treatment step, the FastDNA® Spin kit, the UltraClean™ and PowerSoil™ soil kits and a published manual method based on phenol:chloroform purification, termed Griffiths. *M. bovis* BCG Pasteur spiked samples were extracted by four operators and evaluated using a specific real-time PCR assay. A novel inhibition control assay was used alongside spectrophotometric ratios to monitor the level of inhibitory compounds affecting PCR, DNA yield, and purity. There were statistically significant differences in *M. bovis* detection between methods of extraction and types of environmental samples; no significant differences were observed between operators. Processing times and costs were also evaluated. To improve *M. bovis* detection further, the two best performing methods, FastDNA® Spin kit and Griffiths, were optimised and the ABI TaqMan environmental PCR Master mix was adopted, leading to improved sensitivities.

**Conclusions:**

*M. bovis* was successfully detected in all environmental samples; DNA extraction using FastDNA® Spin kit was the most sensitive method with highest recoveries from all soil types tested. For troublesome faecal samples, we have used and recommend an improved assay based on a reduced volume, resulting in detection limits of 4.25×10^5^ cells g^−1^ using Griffiths and 4.25×10^6^ cells g^−1^ using FastDNA® Spin kit.

## Introduction

Environmental pathogens threaten human, animal and plant health, creating a need for rapid, specific and robust diagnostic methods. For instance, molecular detection of *Mycobacterium bovis* in naturally contaminated soils and animal faeces deposited into the environment [1], [2] has led to an increased interest in the epidemiological significance of environmental reservoirs of *M. bovis* in the persistence of bovine tuberculosis (bTB) in cattle herds and wildlife populations. This is of particular relevance in the United Kingdom, Republic of Ireland, and New Zealand, where wildlife transmission cycles are well established [3], [4], [5] and there is no wildlife test and slaughter policy to remove potentially infectious animals. Mounting evidence suggests that once excreted into the environment, *M. bovis* cells can survive for substantial periods of time (several months to years [1], [6], [7], [8], [9]) with a significant proportion of cells (minimally c. 30%) intact and viable [3], [10], [11], [12]. Historical experiments demonstrate that susceptible cattle can become infected when exposed to naturally or artificially contaminated pasture (reviewed by [12]). Collectively, these data suggest that the environment could act as a significant reservoir of *M. bovis*, which may help explain bTB breakdown persistence in some herds but not others [13].

*M. bovis* cultivation from environmental matrices is problematic as this is an intrinsically slow growing organism (four weeks on selective culture media in optimal conditions), and represents only a small fraction of the estimated 10^10^ total bacterial community per g of soil; *M. bovis* is sensitive to the harsh pre-treatment or decontamination methods necessary to remove competing soil bacteria on culture plates. In addition, *M. bovis* cells are likely to be in an altered physiological state once outside the mammalian host (or culture media), as pathogens can enter a resilient, but quiescent state, in order to survive the biotic and abiotic stresses of the environment, as demonstrated for *Vibrio cholerae* [14]. Approaches such as immunomagnetic capture circumvent the need for cultivation but are currently neither reliable nor suited to high throughput sample screening [15]. We have recently developed a real-time PCR assay for bTB that could be an ideal screening surveillance tool of use for improving farm biosecurity [15]. The reliability of such a test however depends on efficient extraction of *M. bovis* DNA from environmental samples. DNA extraction from soils can be hindered by the presence of humic and fulvic acids, which have similar physico-chemical properties to DNA making the two difficult to separate. Faeces contain biliary salts, urea, haemoglobin and heparin [16] in addition to other compounds, depending on the diet of the animal, which can affect DNA amplification by PCR. The waxy cell wall of mycobacteria, and the possibility of spore formation under conditions of stress [17] may further hinder lysis and DNA recovery.

Published DNA extraction protocols for soils [18], [19] address PCR inhibition to varying extents by including refinement steps such as column chromatography or chemical flocculation, however these methods are laborious, time consuming, expensive and therefore inappropriate for high throughput processing [20], [21], [22].

Here we report a blinded multi-operator randomised trial to evaluate four commercial DNA extraction kits and one previously published manual method for their comparative ability to recover and detect *M. bovis* target DNA in soil and faecal samples. The test kits were Ultraclean™, Powersoil™, QIAamp Stool mini kit, and FastDNA® Spin Kit; the manual method was adapted from the one published by Griffiths [19]. The specific aims were: (i) to measure the analytical sensitivity and the extraction efficiency of these methods in extracting known quantities of *M. bovis* DNA from spiked substrates, (ii) to determine the reproducibility of each method by replication with multiple operators; (iii) to quantify the loss of sensitivity that may be due to carry over of contaminants using a novel inhibition control PCR assay, and (iv) to analyse cost benefits ratio and “hands-on” time for each method. The two methods with the highest analytical sensitivity and reliability were optimised by further protocol development. We conclude by recommending DNA extraction methods towards an optimised real-time PCR assay for quantifying *M. bovis* and similar hard to lyse microorganisms in complex environmental substrates.

## Materials and Methods

### Strains and media

Middlebrook 7H9 broth (BD, Oxford, UK) containing 0.05% Tween 80 (Sigma-Aldrich, St. Louis, MO, USA), was sterilised by autoclaving at 121°C for 20 min. The medium was allowed to cool and was supplemented with OADC enrichment medium (BD, Oxford, UK) prior to inoculation of a single colony of *M. bovis* BCG Pasteur. A 50 mL culture was grown for three weeks, when cells were harvested and filtered through a 30 µm mesh filter, then through a 5 µm filter. Cells were then enumerated by flow cytometry with a CyFlow®space instrument (Partec, Canterbury, UK) using side scatter and fluorescence when stained with 5 µM SytoBC (Molecular probes, Invitrogen, Paisley, UK) for 10 mins in order to get a monodispersed suspension free of large flocs or planktonic micro-colonies, ensuring an accurate serial dilution of the inoculum for spiking. For the enumeration of cells in order to produce genomic DNA standards for real-time PCR quantification, DNA was extracted from an aliquot of the filtered culture with the DNeasy Blood & Tissue Kit (Qiagen Ltd., Crawley, UK). After reading the absorbance at 260 nm with a NanoDrop 1000 spectrophotometer (NanoDrop products, Wilmington, DE, USA) genome equivalents were calculated converting the weight recorded into genomic molecular weight, assuming published genome of strain BCG Pasteur of 2.88×10^9^ Da and the equivalence of one copy of the RD4 deletion target region per cell. Cells used for spiking were also enumerated by calculation of genome equivalents to remove any bias due to enumeration method.

### Sample collection and inoculation with *M. bovis* cells

Five substrates were used, including badger faeces, cattle slurry, and three different soil types. Badger faecal samples were collected from a local badger latrine, and cattle slurry collected from grazing pasture of the same anonymised farm in Warwickshire, UK.

The three soil types (Table S1) were collected from (i) Cryfield (Lat. 52.37042, Lon. −1.55711) (ii) Stockton (Lat. 52.28140, Lon. −1.35938) both in Warwickshire, UK, and (iii) Kilkenny 34 (Lat. 52.88614, Lon. −7.50723) in the Republic of Ireland.

Soils were sieved through 2 mm mesh and allowed to air dry, then were stored at room temperature and faeces were kept at −20°C until testing. All five substrates were confirmed to be PCR negative for *M. bovis* by performing four real-time PCR tests in triplicate on four DNA extractions per sample using the QIAamp Stool Mini kit (Qiagen Ltd., Crawley, UK).

A total of 800 tubes (160 per substrate) were labelled with unique barcodes, randomly selected and filled with 0.5 (±0.2) grams of soil or faeces. For each substrate, 20 tubes were then spiked with 100 µl of each of seven 10-fold dilutions of *M. bovis* to result in 8.5×10^2^ cells g^−1^ to 8.5×10^8^ cells g^−1^; a further 20 tubes were spiked with sterile water. A set consisted of 40 tubes (5 substrates, 8 spikes). Samples were stored at −20°C before processing.

For optimisation,(see results), a total of 224 tubes (64 for Warwick soil and Badger Faeces, 32 for the other substrates) were also labelled with unique barcodes, randomly selected and filled with 0.1 g (±0.1) of substrate. The 7 dilutions of *M. bovis* BCG Pasteur ranged from 4.2×10^2^ cells g^−1^ to 4.2×10^8^ cells g^−1^ in ten-fold dilutions, and each tube was spiked with 20 µl of each dilution or with sterile water prior to storage at −20°C until processing.

### Trial randomisation and blinding

To record details of testing, the 800 barcoded substrate tubes, as well as those with the extracted DNA, were scanned into a PostgreSQL relational database (PostgreSQL Development Group) with a Microsoft Access user interface and managed by an independent database operator. To ensure blinding, information on the substrate type, spiked BCG cell loads, and each stage of matching (sample preparation, extraction, nano-spectrophotometer data for yield and quality of DNA and PCR amplification results) were scanned into the database. Then, identifying marks on the spiked tubes, other than barcodes, were removed with acetone by an independent operator. Replicate sets were randomly mixed and given to each of the four operators for processing with each of the five DNA extraction methods. All operators did the testing at the University of Warwick. Unblinding occurred after all experimental work was completed and data had been entered into the database. A similar approach for randomisation and blinding was adopted for the optimisation assay.

### Trial DNA extraction protocols

The five DNA extraction protocols trialled included four DNA extraction kits and one manual DNA and RNA extraction method. These were: Ultraclean™ (MO BIO, Carslbad, CA, USA); Powersoil™ (MO BIO, Carslbad, CA, USA); QIAamp Stool Mini Kit (Qiagen Ltd., Crawley, UK); FastDNA® Spin Kit for Soil (MP Biomedicals, Solon, OH, USA), and the manual method as previously described for nucleic acid extraction from soils [19], referred to hereafter as the Griffiths method. In all cases, either the manufacturers' instructions or the published protocol were followed with slight modifications: (1) for the FastDNA® Spin Kit, a Precellys®24 (Bertin, Montigny-le-Bretonneux, FR) instrument was used instead of the recommended Fastprep® instrument, to ribolyse samples at 5500 cycles per min for 30 sec in the Lysing Matrix tubes provided. (2) Specimens treated with the Griffiths method were resuspended in 0.5 ml 0.5% CTAB and underwent bead beating with the Precellys®24 homogeniser (Bertin, Montigny-le-Bretonneux, FR) with constant shaking at 5500 cycles per min for 30 sec. (3) The QIAamp Stool Mini Kit procedure included the manufacturer's optional pre-treatment step of heating samples in a water bath at 95°C for 10 min, with a prior modification of pre-filling tubes with glass beads and ASL buffer, and disrupting the cells using a Precellys®24 ribolyser at 5500 cycles per min for 30 sec. (4) For Griffiths and QIAamp Stool Mini Kit O-ring screw cap tubes were prefilled with approximately 0.5 g of 106 µm diameter unwashed glass beads (Sigma-Aldrich, St. Louis, MO, USA) prior to use in the Precellys®24 device at 5500 cycles per min for 30 sec.

### Examination of DNA quality and quantity

Each DNA extract was analysed with a NanoDrop 1000 spectrophotometer (NanoDrop products, Wilmington, DE, USA) to determine DNA concentration and the A260/280, A260/230 and A260/270 nm absorbance ratios. These ratios indicate, respectively, protein, humics and phenolics contamination. To determine total yield per sample the nucleic acid concentration measured with the spectrophotometer at 260 nm was multiplied by 50 (1 OD value = 50 µg/ml) and then by the elution volume specific for each kit.

#### Real-time quantification of *M. bovis*

Extracted DNA was stored at −20°C for at least 12 hrs before processing. Amplification of the specific RD4 region of *M. bovis* in soil and faecal DNA extracts was performed as previously described [15] with the ABI 7500 Fast Real-Time PCR System (Applied Biosystems Inc., CA, USA). All samples were also diluted tenfold in water and 1 µl of diluted extract was subjected to amplification as described above. A subset of DNA extracted with the Griffiths method (85 samples, of which 45 from badger faeces) was also run with the recently marketed 2× TaqMan environmental PCR Master mix (Applied Biosystems Inc, CA, USA) using the same conditions as previously stated.

#### DNA standards and interpretation of real-time assay

Genomic DNA obtained from a filtered culture of *M. bovis* BCG was used to generate a standard curve of genomic equivalents for the real-time PCR over a dilution range from 845000 to 20 units per PCR reaction. DNA standards were run in triplicate on each quantitative plate. Samples were considered positive if each triplicate Ct value was above the baseline with the auto threshold set on default for the instrument. Samples with <3 positive Ct values were rerun, and then again if the number of positive Ct values remained <3. Samples with <3 positive Ct values on three runs were thus classed as negative.

### Recovery, analytical sensitivity and theoretical detection limit

Recovery was calculated as the number of cells detected across the four highest spikes compared with the spike titre, expressed as percentage (Table 1). The percentage of all samples at the specified spike dilution testing positive across operators was taken as analytical sensitivity. This gave the lowest spike at which all four operators could detect at least one true positive sample (Table 2). The theoretical detection limit (TDL) of the methods was considered, i.e. the minimal inoculum (cells) necessary to detect 1 genome copy (cell) (Table 2). This is dependent on the size of the sample, on the dilution factor used in the PCR reaction and on the volume in which the DNA is eluted following extraction. The TDL was calculated from:
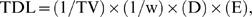
where TV is the volume (µl) of the template used in the PCR reaction, w is the weight of the sample (g), D is the dilution factor and E is the elution volume of the kit (µl).

**Table 1:** Recovery for all sample types.

Extraction method							
						modified	modified
Sample type	Griffiths 0.5 g	Powersoil™ 0.5 g	Ultraclean™ 0.5 g	FastDNA® Spin kit 0.5 g	QIAamp Stool kit 0.5 g	Griffiths 0.1 g	FastDNA® Spin Kit 0.1 g
Badger faeces	0.00 (0.00–0.00)	0.00 (0.00–0.00)	0.00 (0.00–0.00)	0.06 (0.00–0.12)	0.00 (0.00–0.00)	10.87 (5.73–14.67)	21.48 (13.94–48.82)
Badger faeces (10× diluted)	0.05 (0.00–0.12)	0.00 (0.00–0.00)	0.00 (0.00–0.00)	0.00 (0.00–0.05)	0.00 (0.00–0.00)	n.a.	n.a.
Cow slurry	0.19 (0.13–0.28)	0.00 (0.00–0.01)	0.00 (0.00–0.00)	0.07 (0.00–0.17)	0.00 (0.00–0.00)	n.a.	11.48 (8.72–23.43)
Kilkenny soil	18.72 (9.13–63.26)	23.92 (8.45–32.65)	1.55 (0.8–4.41)	85.04 (30.25–100)	12.53 (11.13–16.11)	n.a.	51.05 (16.12–62.72)
Stockton soil	6.97 (3.37–21.42)	8.82 (4.58–9.93)	0.89 (0.58–1.8)	23.08 (10.51–30.2)	1.86 (0.68–3.95)	n.a.	30.79 (6.57–43.96)
Warwick soil	16.23 (6.7–21.28)	18.07 (10.3–26.93)	10.51 (5.83–18.8)	79.79 (49.31–100)	9.99 (7.89–12.03)	2.91 (0.32–9.59)	49.16 (27.58–73.54)

The recovery (percentage) shown is the median value of the top 4 spikes, interquartile range values are presented in brackets. n.a.: not available.

**Table 2:** Cost efficiency analysis.

	Costs (£)	Hands on time (hrs)	Theoretical detection limit (cells)	Soil analytical sensitivity^1^ (cells g^−1^)	Cost - efficiency score	Faeces analytical sensitivity^1^ (cells g^−1^)	Cost - efficiency score
QIAamp stool kit	4.78	5.18	4×10^2^	1.83×10^6^	30	8.5×10^8^	43
Powersoil™	4.65	5.01	4×10^2^	8.5×10^8^	28	8.5×10^8^	42
Ultraclean™	3	4.28	1×10^2^	3.95×10^5^	17	4.25×10^8^	26
FastDNA® Spin Kit	4.05	4.57	2×10^2^	8.5×10^4^	20	1.9×10^7^	29
Griffiths	2.78	2.51	1×10^2^	8.5×10^4^	14	1.9×10^6^	17
Modified FastDNA® Spin Kit 0.1 g	4.05	4.57	1×10^3^	4.25×10^5^	23	4.25×10^6^	27
Modified Griffiths 0.1 g	2.78	2.51	5×10^2^	4.25×10^6^	18	4.25×10^5^	16

Lower scores indicate greater cost-effectiveness. ^1^Data are expressed as geometric means of either the three soil types or the two faecal types analytical sensitivities, respectively.

### Construction of an inhibition control plasmid

In order to assess inhibition by contaminants co-extracted with the DNA, a synthetic construct was developed containing a green fluorescent protein (GFP) sequence flanked by *M. bovis* RD4 region primer sites, which was cloned into TOPO pCR®2.1 plasmid (Invitrogen, Paisley, UK) according to manufacturers instructions, to give RD4-GFPpCR®2.1. The fusion was produced synthetically by annealing the two oligonucleotides RD4-GFP-S and RD4-GFP-AS ( ^5′^-TGTGAATTCATACAAGCCGTAGTCGAAGATACCCAGATCATATGAAACAGCATGACTTTTTCAAGAGTGCCATGCCCGAAGGTTAGCAATTTCTCAGTAACGCTACGGGA-^3′^ and ^5′^CCCGTAGCGTTACTGAGAAATTGCTAACCTTCGGGCATGGCACTCTTGAAAAAGTCATGCTGTTTCATATGATCTGGGTATCTTCGACTACGGCTTGTATGAATTCACAA-^3′^ , respectively). RD4-GFP-S started from the 5′ end with the *M. bovis* RD4 forward primer sequence directly next to residues 61–120 of GFP (sequence acc. No. M62653) and was followed by the reverse complement sequence of the RD4 reverse primer. RD4-GFP-AS was the reverse complement of the previous. An additional adenosine (A) residue had been added to the sequences at the 3′ ends, to facilitate TA cloning into vector pCR®2.1. Annealing was performed by boiling the oligonucleotides (0.1 µg each) in 20 µl annealing buffer (10 mM Tris-HCl pH 8.5; 5 mM MgCl_2_) and then cooling the mix to room temperature.

### Inhibition control assay

The RD4-GFPpCR®2.1 plasmid was added to a subset [one replicate panel of each substrate, for the 4 kits (25%) and three replicate panels of each substrate for Griffiths (75%)] of samples to take into account any PCR inhibition thought to result from residual contaminants. The probe for the GFP in the inhibition control assay contained ‘locked’ nucleotide bases (LNA) which increase the stability of hybridization to the target sequence [23], [24].

Each reaction contained: 12.5 µl of Applied Biosystems 2× TaqMan universal PCR Master mix, 1 µl of primer *M. bovis* F ^5′-^TGTGAATTCATACAAGCCGTAGTCG-^3′^ , 1 µl of primer M. bovis R ^5′-^CCCGTAGCGTTACTGAGAAATTGC-^3′^ , 1 µl of probe ^5′-^JOE-ATATGAAA+CAG+CATGA+CTTT—BBQ-^3′^ (+ = LNA base), 1 µl of RD4-GFPpCR®2.1 plasmid(2.7 ng/µl ), 2.5 µl of filter sterilised Bovine Serum Albumin (10 mg/ml) (Sigma-Aldrich, Dorset, UK) and 5 µl of filter sterilised MonoQ water. For each sample, reactions were conducted in triplicate and 1 µl of extracted DNA was added to each plate well except for the triplicate no inhibition control (NIC) wells which had sterile water added. The difference in Ct values of the samples compared to NIC was referred to as Delta Ct (ΔCt). Inhibition was detected when ΔCt values were above zero, and when an effect was observed on RD4 detection, with negligible to moderate inhibition up to 1 ΔCt. A ΔCt value of 1 would theoretically predict a 2 fold decrease in RD4 detection, whilst higher ΔCt values would account for more dramatic decreases.

### Statistical Data Analysis

Quantitative recovery of *M. bovis* cells was calculated as the percentage of cells detected compared to that spiked for each sample. Differences in quantitative recovery, DNA yield and spectrophotometric ratios were analysed using the non-parametric Kruskal-Wallis analysis of variance, with more detailed pairwise analyses performed using the Wilcoxon rank sum test with a Bonferroni correction. Smile plots were produced using the Wilcoxon rank sum test with a Holland correction [25]. The cut-off *p* value (0.05) and the Holland correction factor (adjusted cut-off *p* value 0.0253) are shown on the smile plots. The relative values for the spectrophotometric A260/230 ratio are expressed as a proportion, i.e. the difference in the median of the ratios for the two methods divided by the median of the ratio for the first method. All statistical analyses were performed using STATA/IC v. 11.1 (StataCorp LP, College Station, TX, U.S.A.).

### Time and costs of the DNA extraction protocols

Cost-efficiency analysis of the DNA extraction methods was performed by measuring the average time required to complete 20 samples starting at the time of weighing the aliquot tubes to the moment when DNA extracts were put into storage at −20°C. Commercial purchase costs of kits and/or reagents (chemicals, enzymes, and disposable items including microfuge tubes for the manual method) were obtained from manufacturers and are expressed per sample. These data were compared to the analytical sensitivity of each test to give a comparative score of cost-efficiency (CE), where CE = cost per sample×log_10_ analytical sensitivity of the method (expressed as the geometric mean of the analytical sensitivities of soils or faeces) (Table 2).

## Results

### Comparison of DNA extraction methods for analytical sensitivity and extraction efficiency

**Figure 1:**
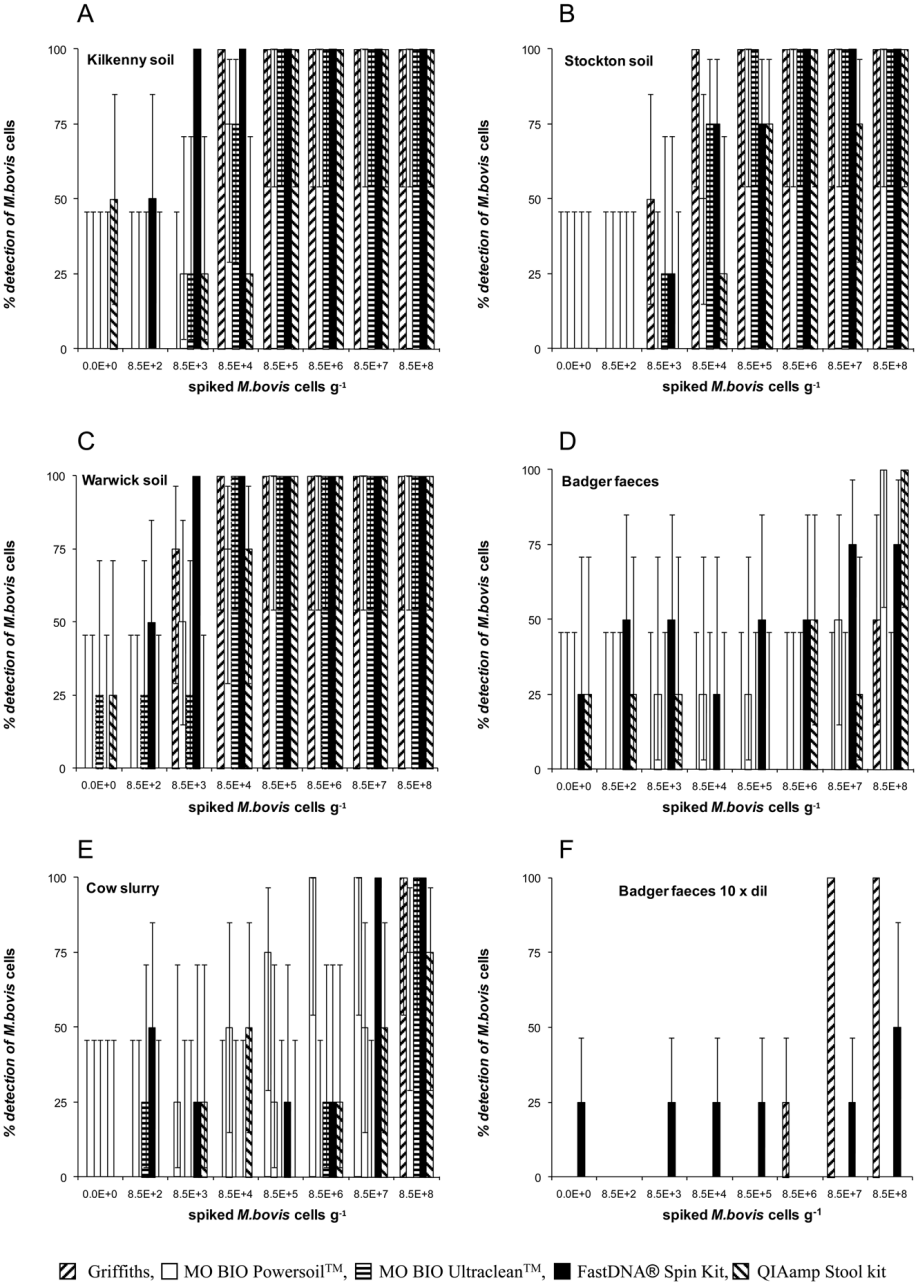
Analytical sensitivities of the DNA extraction trials. Percentage detection of positive soil (A, B, C) and faecal samples (D, E, F) spiked with *M. bovis* BCG at a range of cell counts per sample with different kits. (F) Represents amplification from 1 in 10 diluted template. Error bars indicate 95% binomial confidence intervals.

**Figure 2:**
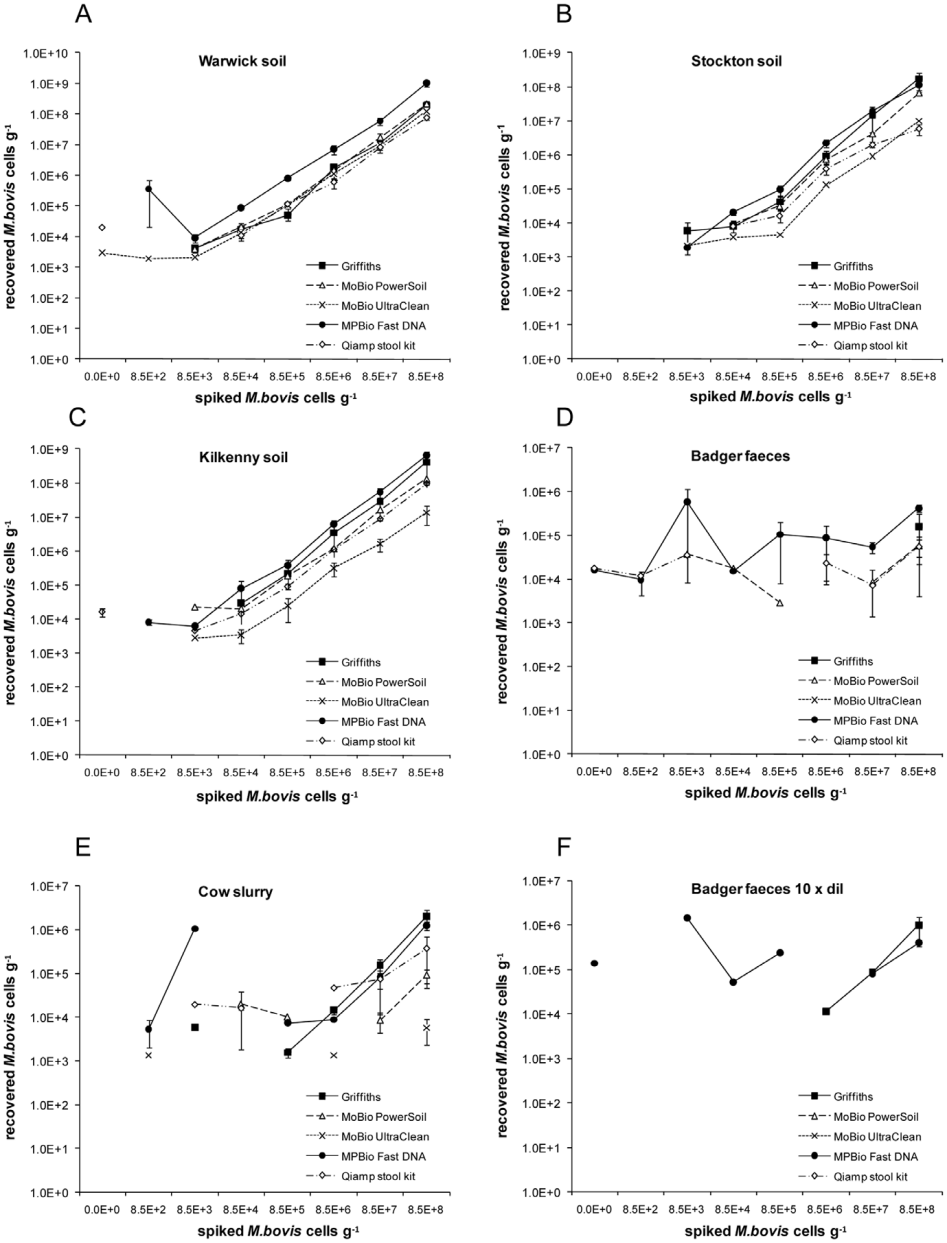
Recoveries of the DNA extraction trials. *M. bovis* BCG detected from three soils: Warwick (A), Stockton (B) and Kilkenny (C) seeded with known numbers of BCG cells. *M. bovis* BCG detected from badger faeces (D, F) and cow faeces (E) seeded with known numbers of BCG cells. (F) represents amplification from 1 in 10 diluted template. Note the log scale for recovered BCG. Data points are means of any positive results obtained by any of the operators. Error bars represent ±1 standard error of the mean.

Analytical sensitivity is expressed as the spike titre at which 100% of operators detected *M. bovis* cells (Fig. 1). Recovery was determined as the number of cells detected across the four highest spikes compared with the spike titre (Table 1, Fig. 2). All five methods of extraction performed least well on faeces. In comparisons between the three soil types there were significant differences in test sensitivity (Kruskal Wallis, *p*<0.01), differences between methods of extraction, and between substrates (Kruskal Wallis, *p*<0.01).

No statistically significant differences were observed between the four operators' results, when extraction methods or substrate type were compared (Kruskal Wallis, *p*>0.05).

Across sample types and methodologies, a high recovery tended to correlate with a good analytical sensitivity (Table 1, Fig. 1).

The method with highest recovery and analytical sensitivity varied dependant on the soil type. FastDNA® Spin Kit performed very well with the optimal recovery (100%) and the lowest analytical sensitivity at 8.5×10^3^ on Kilkenny and Warwick soils (Wilcoxon rank sum, *p*<0.05).

Griffiths produced the highest recovery 18% (4–42) and lowest analytical sensitivity (8.5×10^4^ cells g^−1^) on Stockton, a soil higher in clay and organic matter content (Table 1) and which gave the lowest recovery using all five methods. Ultraclean™ performed the least well in terms of recovery (Wilcoxon rank sum, *p*<0.01) (Table 1).

On both cow and badger faeces DNA, recovery was poor (<1%) irrespective of method: detection either failed or the analytical sensitivities were substantially higher than for soils (Table 1, Fig. 1). Noteably, Ultraclean™ failed to detect at any spike on badger faeces (Table 1, Fig. 1).

To attempt to improve sensitivity, all extracted DNA were also diluted tenfold before testing by real-time PCR. Dilution of extracted DNA improved sensitivity only for badger faeces (Table 2, Fig. 1).

Based on these analytical sensitivity and % recovery data, the Griffiths and the FastDNA® Spin Kit proved to be the two best performing methods.

### Further method development for Griffiths and FastDNA® Spin Kit

**Figure 3:**
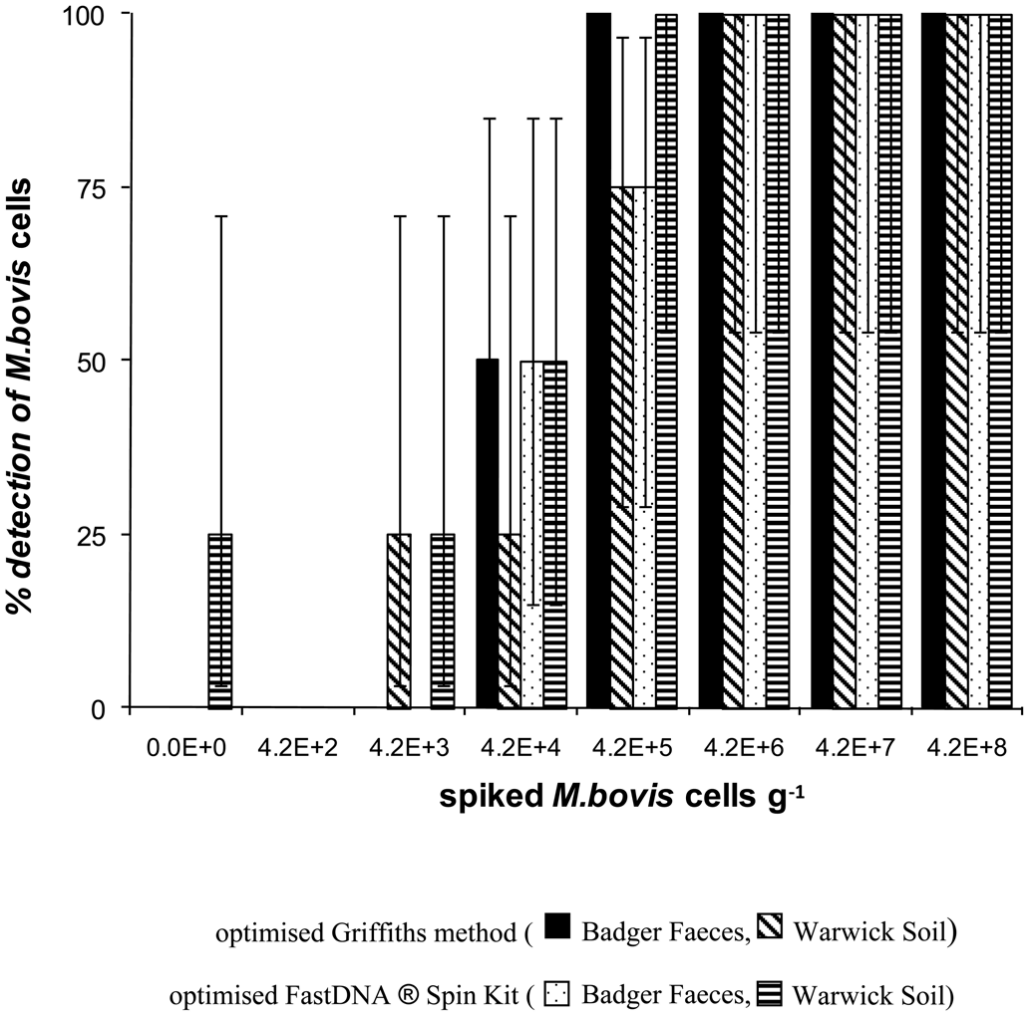
Further method development. Percentage detection by four operators of positive soil and faecal samples spiked with BCG at a range of cell counts with the optimised Griffiths method and with the optimised FastDNA® Spin Kit. Error bars indicate 95% binomial confidence intervals.

The Griffiths and the FastDNA® Spin Kit were modified to improve analytical sensitivity and recovery and to reduce contamination. The sample was reduced from 0.5 g to 0.1 g (in combination, for the Griffiths method only, with a double ribolysis step and a 2 hrs DNA precipitation in PEG). On the badger faecal samples, this reduction resulted in an improved analytical sensitivity of both methods (Wilcoxon rank sum, *p*<0.01) (Fig. 3): the “modified” Griffiths gave 100% detection by all operators at spike 4.2×10^5^ cells g^−1^ compared to detection of 0% at all spikes using the original Griffiths method on the 0.5 g samples. Recovery and sensitivity were also improved using the “modified” FastDNA® Spin Kit; when reducing the sample to 0.1 g badger faeces, 100% detection was achieved at spike 4.2×10^6^ cells g^−1^ (three out of four operators detected at a spike of 4.2×10^5^ cells g^−1^). For soil, reduction of the sample size to 0.1 g did not result in uniform improvements. For Warwick soil, the modifications to both methods resulted in lower recoveries and higher analytical sensitivities (Wilcoxon rank sum, *p*<0.01). The modified FastDNA® Spin Kit was also applied to Kilkenny and Stockton soils and to cow faeces. Improved recovery and sensitivity were observed for cow faeces, whereas reducing the sample size of soils resulted in improved sensitivity for Stockton soil only, but did not improve the recovery from any soils.

### Specificity

Three DNA extraction methods gave false positive counts in samples with no BCG added, FastDNA® Spin Kit (5%), QIAamp Stool kit (20%), and the Ultraclean™ kit (5%) (Fig. 2), indicative of cross-contamination. In addition, using FastDNA® Spin Kit with a reduced sample size still gave rise to false positives tests (15%). For FastDNA® Spin Kit, observations showed tube leakage was responsible and was overcome by the manufacturer replacing Lysing Matrix tubes supplied with the kits. Subsequent testing revealed no false positives (data not shown).

### Assessment of inhibition

**Figure 4:**
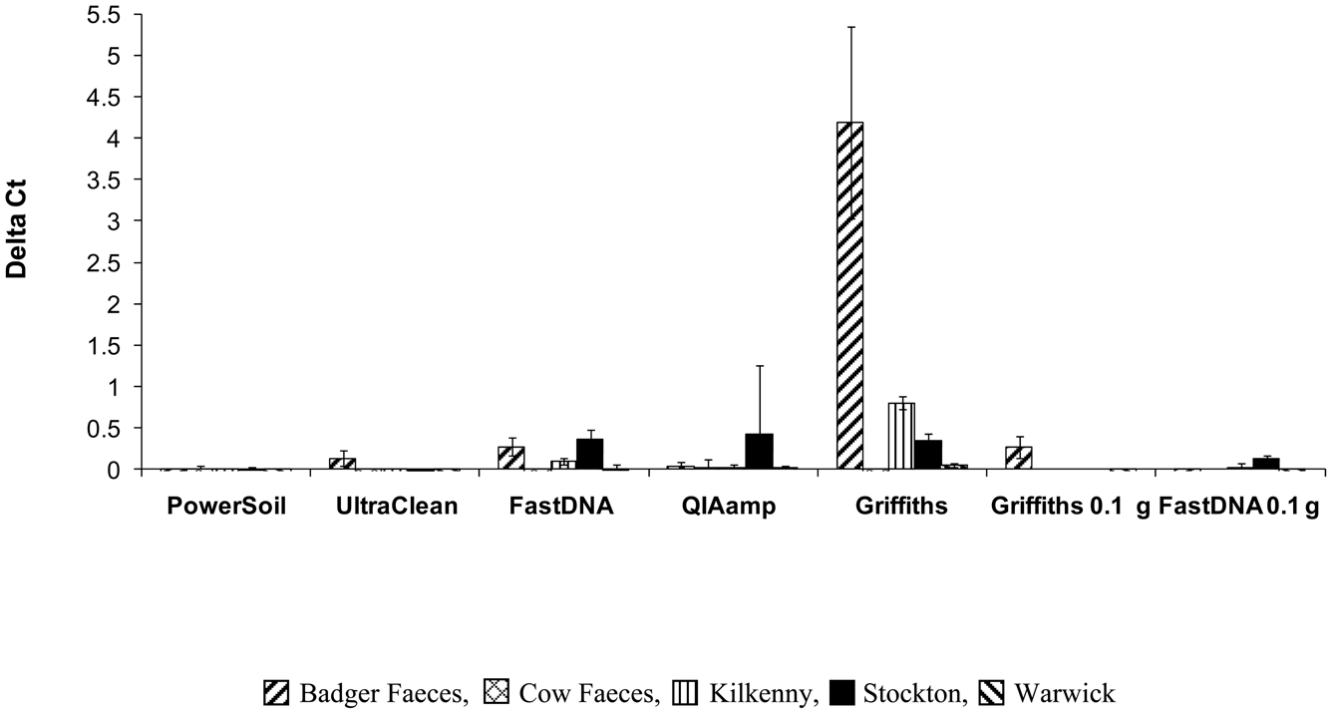
Assessment of inhibition. Inhibition assay with plasmid RD4-GFPpCR®1.2. ΔCt values presented for all methods tested by one operator. Error bars represent ±1 standard error of the mean.

Addition of an inhibition control enabled quantification of contaminants in extracted DNA (Fig. 4). Control reactions were performed separately to the RD4 assay to avoid primer competition for the same target sequences in extracted DNA. Variations of inhibition expressed by the ΔCt value were observed between methods and between sample types (Kruskal Wallis, *p*<0.01). The largest inhibition observed was in DNA extracted using the non- modified Griffiths method, badger faeces being particularly affected (Wilcoxon rank sum, *p*<0.05). When 0.1 g vs 0.5 g sample material was used, inhibition was clearly reduced for faecal samples extracted using both Griffiths and with the FastDNA® Spin Kit.

A further reduction of inhibition due to contaminants co-extracted in the Griffiths method was achieved using the recent commercially available 2× ABI TaqMan environmental PCR master mix. A small test on all DNA extracted from badger faeces with Griffiths improved sensitivity to 75% detection at the spike of 8.5×10^5^ cells g^−1^ compared to no detection for neat or diluted badger faecal extracts of the same sample amplified with the conventional master mix.

### Quality of DNA extracted with the different methods

The DNA absorbance ratios are a useful indicator of contamination of DNA by humics (A260/230, optimal 2), phenolics (A260/270, optimal 1.2) and proteins (A260/280, optimal 1.8). Absorbance ratios were determined for all DNA extracted and these were analysed with the Kruskal–Wallis test where significant differences were found between sample types, operators and methods (*p*<0.05) in all cases (Table S2). For all ratios, values were consistently lower than optimal, indicating varying level of contaminants.

**Figure 5:**
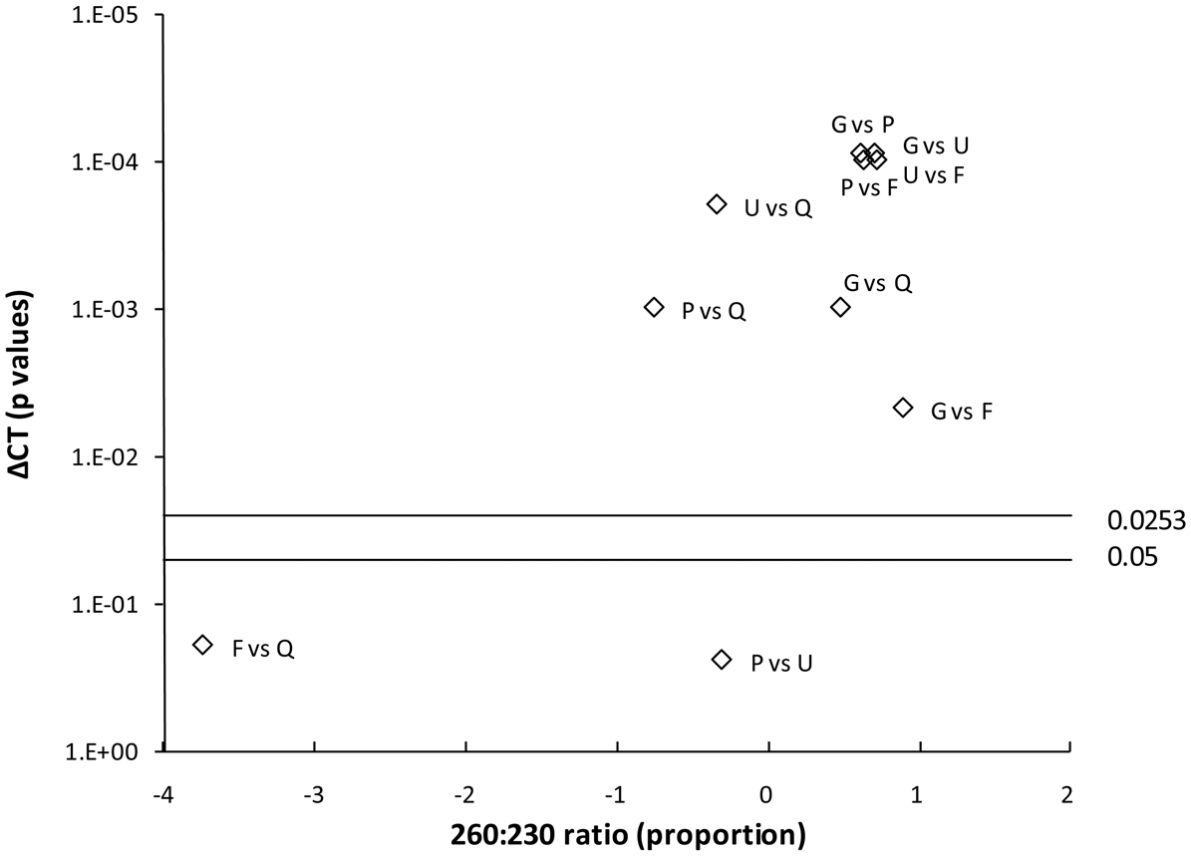
Linking inhibition to DNA purity. Smile plot of the pairwise comparison between each method for ΔCt values (*p* values, Wilcoxon rank sum test) against the A260/230 ratio (expressed as proportion of the values for the two methods). The cut-off *p* value (0.05) and the Holland correction factor (adjusted cut-off *p* value 0.0253) are shown. G: Griffiths; P: Powersoil™; U: Ultraclean™; F: FastDNA® Spin kit; Q: QIAamp Stool kit.

The ratios for the Griffiths method did not indicate significant phenol or humic contamination; the A260/230 ratio was significantly higher and closest to the optimal compared to the other extraction methods (Wilcoxon rank sum, *p*<0.01, Table S2). The smile plot (Fig. 5) indicates a correlation between high A260/230 ratios and ΔCt values, the latter being a clear indication of inhibition. For the other extraction methods, there is no clear correlation between suboptimal A260/230 ratios and inhibition. The Griffiths method gave significantly higher yield compared to the other extraction methods but which may be due to co-extraction of RNA (Table S2).

### Cost benefit analysis

The fastest and cheapest method was the Griffiths although the precipitation step was excluded from the recorded hands-on time (Table 2). The cost benefit analysis relates cost to analytical sensitivity and again Griffiths gave the best score followed by Ultraclean™ and FastDNA® Spin Kit. It should also be noted that all methods require initial purchase of additional equipment, e.g. the Vortex adaptor (for MO BIO kits) and Precellys®24 homogeniser (for all other methods) which should be added to the costs reported here (Table 2). For the Griffiths, additional costs should also be considered, which could be incurred for safe utilisation and disposal of phenol.

## Discussion

A trial involving comparison of five DNA extraction methods was performed by multiple operators for molecular detection of environmental *M. bovis* in soils and faeces. Statistical differences were not detected between operators within any of the extraction methods; however there were clear differences in test performance. The trial showed remarkable differences between substrate types (soils and faeces) and DNA extraction methods. Recovery and analytical sensitivities were used as indicators of performance. Analytical sensitivities were similar to other studies using real-time PCR to determine recovery of other pathogenic microorganisms from environmental matrices [26]. The Griffiths manual method and the FastDNA® Spin Kit were the most promising for provision of a sensitive and reliable environmental assay. Optimisation of the sample size with reduction to 0.1 g significantly improved performance of these methods for faecal samples. Reducing the sample size is consistent with previous studies showing that a small sample size allows efficient extraction from difficult samples [27], in part due to the reduction in amounts of contaminants co-extracted. The use of multiple operators to measure repeatability provided a more robust trial compared to previous studies that involved only single operators [28], [29], [30].

False positives were obtained with some of the kits, in the case of FastDNA® Spin Kit this may have been due to tube leakage; concomitantly, for this kit the manufacturer has developed new leak proof tubes replacing the original Lysing matrix tubes. In addition, all kits use a spin column for purification of DNA and during centrifugation cross-contamination can occur due to aerosol formation if the spin columns are not placed firmly enough into the collection tubes during the various centrifugation steps. This problem has also been observed in other studies on DNA extraction [31], [32].

The development of an inhibition control was a very valuable addition to the assay, providing an accurate indication of the impact of contaminants in extracted DNA on analytical sensitivities. Absorbance ratios failed to provide a reliable indication of contaminated extracts, as illustrated by the Griffiths method, which despite showing high ΔCt values, gave the best absorbance values.

The accuracy of the inhibition control assay relates to the use of identical PCR target sequences in contrast to other published methods where different PCR targets are tested on the same samples [29], [33], [34], [35]. Use of the same target did require a separate assay for detection of inhibition to avoid primer competition for target. We hypothesize that further optimisation of our assay could lead to a simultaneous use in the same reaction. Ultimately, the use of the inhibition control also allows identification of such false negative results, allowing for re-testing, and allocation of unresolved status in data analysis. The inhibition control assay revealed moderate to strong inhibition in some soil and faecal extracts. For badger faeces, inhibition could be reduced by diluting template DNA, although this did reduce sensitivity. A potentially better solution for reducing inhibition, identified by our preliminary test, was to adopt an environmental master mix which resulted in better sensitivities without the need for dilution for badger faeces. Furthermore, we demonstrated that using the Griffiths method or the FastDNA® Spin Kit, the limit of detection could be improved in faeces by reducing the amount of sample processed.

In conclusion, we demonstrate the considerable effort is required to ensure reliability and sensitivity of molecular assays to quantify pathogens in complex environmental samples. We recommend the use of either the Griffiths method or the FastDNA® Spin Kit, in conjunction with an inhibition control, and 2× TaqMan environmental PCR Master mix for extraction of DNA from soil and faeces. In addition, testing a smaller sample (0.1 g) of faecal material should help to further reduce inhibition and improve sensitivity. Molecular detection of *M. bovis* in non-invasive environmental samples, such as soils and excreted host faeces, will facilitate the study of the numerical and spatial distributions of *M. bovis* in the environment. Hopefully this will aid in bTB epidemiological surveillance of animal populations and farms.

## Supporting Information

Table S1 Soil characteristics.
(XLSX)

Table S2 Nucleic acids absorbance ratios and yield.
(XLSX)
